# Retraction: Cultivation and interpretation of students' psychological quality: vocal psychological model

**DOI:** 10.3389/fpubh.2023.1221608

**Published:** 2023-05-24

**Authors:** Frontiers Editorial Office

The journal retracts the article cited above.

Following publication, the publisher has found evidence of peer review manipulation. As the scientific integrity of the article cannot be guaranteed, and adhering to the recommendations of the Committee on Publication Ethics (COPE), the publisher therefore retracts the article.

This retraction was approved by the Chief Editors of Frontiers in Public Health and the Chief Executive Editor of Frontiers.

